# Re-defining the Gut Heart Axis: A Systematic Review of the Literature on the Role of Gut Microbial Dysbiosis in Patients With Heart Failure

**DOI:** 10.7759/cureus.34902

**Published:** 2023-02-12

**Authors:** Darshi Desai, Aditya Desai, Aneeque Jamil, Denise Csendes, Sai D Gutlapalli, Keerthana Prakash, Kiran M Swarnakari, Meena Bai, Mohana P Manoharan, Rabab Raja, Safeera Khan

**Affiliations:** 1 Internal Medicine, California Institute of Behavioral Neurosciences & Psychology, Fairfield, USA; 2 Internal Medicine, University of California Riverside School of Medicine, St. Bernardine's Medical Center, San Bernardino, USA; 3 Medicine, California Institute of Behavioral Neurosciences & Psychology, Fairfield, USA; 4 Medicine, All Saints University School of Medicine, Roseau, DMA

**Keywords:** intestinal microbiome, intestinal dysbiosis, dysbiosis, gut microbiome, cardiac failure, chronic heart failure

## Abstract

Heart failure (HF) contributes to the cardiovascular health burden worldwide. Patients with heart failure have been recently studied to possess unique changes in the gut microbiome that affect immune homeostasis and metabolism. In this systematic review of the literature, we aim to identify the impact of gut dysbiosis on heart failure. We used Preferred Reporting Items for Systematic Reviews and Meta-Analyses (PRISMA) 2020 guidelines to conduct our systematic review. We searched the literature on databases such as PubMed, PubMed Central (PMC), Medline, and ScienceDirect. Ten articles were included for review. There were significant differences in the gut microbiome composition in heart failure. Relative abundance of *Ruminococcus gnavus, Escherichia Shigella, Streptococcus sp, Veillonella sp, and Actinobacteria, *and relative depletion of *Eubacterium,* *Prevotella*, *Faecalibacterium, SMB53,* and *Megamonas. *The composition varied according to age, heart failure stage, and decompensation level. The composition remained unaltered with ejection fraction. There was an increased expression of genes responsible for the metabolism of amino acids, carbohydrates, choline trimethylamine-lyase (TMA-lyase), lipopolysaccharide (LPS) biosynthesis, tryptophan, and lipid metabolism. The resultant changes affected the levels of metabolites, such as trimethylamine N-oxide (TMAO), indoxyl sulfate (IS), and LPS, and inflammatory markers in the feces and plasma, which contributed to heart failure. These biomarkers of heart failure could serve as targets for the prevention and treatment of heart failure. Patients with heart failure harbor a unique constellation of gut microbiota that affect the pathogenesis of heart failure. Further studies are needed to understand the causal relationship between dysbiosis and heart failure.

## Introduction and background

Heart failure (HF) is a major public health burden worldwide. It has been estimated that about 6.5 million people suffer from chronic heart failure in the United States [1]. It is an end-stage consequence due to several etiologies that cause structural and/or functional damage to the heart. Depending on the ejection fraction, heart failure is classified as systolic (reduced ejection fraction) and diastolic (preserved ejection fraction). Regardless of the etiology and type, heart failure is associated with systemic inflammation, endotoxemia, and oxidative stress. These processes eventually affect the progression, severity, and, thereby, the prognosis of heart failure. Recently, the gut microbiome has been identified as a major influencer in the pathogenesis of chronic heart failure due to its varied effects on the body, particularly the immune system. This interaction between the gut and the heart is known as the gut-heart axis.

The human body hosts a wide array of microbial species, known as the human microbiome, organized into distinct groups such as the oral, vaginal, gastrointestinal, and skin communities [2-6]. They contribute to overall human homeostasis and disease pathogenesis. The composition of the human microbiome has been studied by culture-independent analyses using newer technology [6]. The alteration of the composition of these communities, referred to as dysbiosis, is implicated in the causation of numerous diseases [2]. The host-associated communities have also been documented to increase host susceptibility to inflammatory bowel disease [7], type 1 diabetes mellitus [8], allergies [9], and cancer [10].

The gut microbiome has been widely studied recently because of its pertinent role in maintaining the immune system [11-16], and the possibility of influencing other organs and systems in the body such as the cardiovascular, renal [17], central nervous [18], musculoskeletal [19], endocrine [20,21], and pulmonary systems [17]. There is growing interest in evaluating an association between intestinal microbial dysbiosis and the development of cardiovascular diseases, particularly atherosclerosis and chronic heart failure. Recent studies have documented the microbial metabolism of dietary phosphatidylcholine into pro-atherosclerotic metabolite trimethylamine N-oxide (TMAO) that could contribute directly or indirectly to cardiovascular outcomes such as coronary artery disease and heart failure in patients with intestinal dysbiosis [22].

Despite a growing interest in this field, the exact relationship between the gut microbiome and heart failure still remains understudied. So far, only limited small-scale studies have been conducted to identify unique changes in the gut microbial composition and metabolic profiles in heart failure patients. Although studies have demonstrated that heart failure patients harbor a distinct set of microbes, whether these changes are a causative factor or a consequence of heart failure is unknown. It is crucial to identify a causal relationship between gut dysbiosis and heart failure and thereby discover specific fecal biomarkers that could serve as therapeutic targets to prevent and treat heart failure.

This systematic review of the literature describes the association and role of dysbiosis in patients with chronic heart failure. Further studies are warranted to demonstrate a temporal relationship between dysbiosis and heart failure, if any exists.

## Review

Methods

We conducted this systematic review strictly adhering to the Preferred Reporting Items for Systematic Reviews and Meta-Analyses (PRISMA) 2020 guidelines [23]. Our study aimed at identifying an association, if any exists, between intestinal microbial dysbiosis and chronic heart failure.

Literature Search Strategy

We thoroughly searched four databases - Pubmed, PubMed Central (PMC), Medline, and ScienceDirect - for articles that studied an association between intestinal microbial dysbiosis and chronic heart failure between January 1, 2022, and March 1, 2022. A systematic search strategy using medical subject headings (MeSH) terms and keywords was developed to extract all the relevant articles for analysis. The keywords included "chronic heart failure", "heart failure", "cardiac failure", "gut microbiome", "dysbiosis", "intestinal dysbiosis", and "intestinal microbiome". Using the Boolean method, we used keywords and the MeSH strategy to retrieve all the pertinent articles from the databases. The detailed search strategy for the databases has been stated Table 1. 

**Table 1:** Detailed literature search strategy

Database	Strategy	Number of results before inclusion/ exclusion criteria	Results after inclusion/ exclusion criteria (studies from last 10 years, adult population)
PubMed, PMC, Medline	(((gut dysbiosis) OR (gut microbiome) OR (intestinal dysbiosis) OR (microbiome)) AND ((chronic heart failure) OR (heart failure) OR (cardiac failure))	370	62
	( "Dysbiosis/complications"[Mesh] OR "Dysbiosis/diagnosis"[Mesh] OR "Dysbiosis/etiology"[Mesh] OR "Dysbiosis/microbiology"[Mesh] OR "Dysbiosis/pathology"[Mesh] OR "Dysbiosis/physiology"[Mesh] OR "Dysbiosis/physiopathology"[Mesh] ) AND ( "Heart Failure/complications"[Mesh] OR "Heart Failure/diagnosis"[Mesh] OR "Heart Failure/diagnostic imaging"[Mesh] OR "Heart Failure/epidemiology"[Mesh] OR "Heart Failure/etiology"[Mesh] OR "Heart Failure/pathology"[Mesh] OR "Heart Failure/physiology"[Mesh] OR "Heart Failure/physiopathology"[Mesh]	16	2
	(( "Dysbiosis/complications"[Mesh] OR "Dysbiosis/diagnosis"[Mesh] OR "Dysbiosis/etiology"[Mesh] OR "Dysbiosis/microbiology"[Mesh] OR "Dysbiosis/pathology"[Mesh] OR "Dysbiosis/physiology"[Mesh] OR "Dysbiosis/physiopathology"[Mesh]) AND ((chronic heart failure) OR (heart failure) OR (cardiac failure)))	27	5
	((gut dysbiosis) OR (gut microbiome) OR (intestinal dysbiosis) OR (microbiome)) AND ( "Heart Failure/complications"[Mesh] OR "Heart Failure/diagnosis"[Mesh] OR "Heart Failure/diagnostic imaging"[Mesh] OR "Heart Failure/epidemiology"[Mesh] OR "Heart Failure/etiology"[Mesh] OR "Heart Failure/pathology"[Mesh] OR "Heart Failure/physiology"[Mesh] OR "Heart Failure/physiopathology"[Mesh] )	100	26
ScienceDirect	(((gut dysbiosis) OR (gut microbiome) OR (intestinal dysbiosis) OR (microbiome)) AND ((chronic heart failure) OR (heart failure) OR (cardiac failure)) AND ("adults"))		679

Articles were limited to original observational studies. Additional inclusive criteria included papers from the last ten years, papers published in the English language, papers focusing on the population with age 19 years and above, and papers relevant to the question. We excluded papers discussing population with age 18 years and below, unpublished literature, and gray literature.

Data Extraction

The articles were screened by title, abstract, and full text for relevance by two authors independently. The data was extracted on an excel sheet. The information extracted from these articles included the year of publication, sample size, demographics of the patients (age), left ventricular ejection fraction (LVEF) % of the chronic heart failure (CHF) patients, inclusion criteria, results, and conclusion. In case of discrepancy between the reviewers, opinion was taken from the third author, and conflicts were resolved by mutual understanding considering the inclusion and exclusion criteria of the review.

Quality Appraisal Of Studies

We used the Joanna Briggs Institute (JBI) tool for the quality appraisal of the included articles. The score of each criterion was reported as "Yes", "No", or "Unclear". The articles that fulfilled >70% of the criteria were finally included for detailed review. The detailed quality check based on the criteria using the JBI tool is described in Table 2.

**Table 2:** Quality appraisal of the included studies N/A - not applicable

Author	Criteria for inclusion?	Study subjects and the setting?	Exposure measured in a valid and reliable way?	Objective, standard criteria used for measurement of the condition?	Confounding factors?	Strategies to deal with confounding factors?	Outcomes measured in a valid and reliable way	Appropriate statistical analysis?
Beale et al. [24]	Yes	Yes	N/A	Yes	Yes	Yes	Yes	Yes
Wang et al. [25]	Yes	Unclear	N/A	Yes	Yes	Yes	Yes	Yes
Hayashi et al. [26]	Yes	Unclear	N/A	Yes	Yes	Yes	Yes	Yes
Yuzefpolskaya et al. [27]	Yes	Yes	N/A	Yes	Unclear	Unclear	Yes	Yes
Cui et al. [28]	Yes	Unclear	N/A	Yes	Yes	Yes	Yes	Yes
Katsimichas et al. [29]	Yes	Yes	N/A	Yes	Yes	Yes	Yes	Yes
Hayashi et al. [30]	Yes	Yes	N/A	Yes	Yes	Yes	Yes	Yes
Kamo et al. [31]	Yes	Unclear	N/A	Yes	Yes	Yes	Yes	Yes
Luedde et al. [32]	Yes	No	N/A	Yes	Yes	Yes	Yes	Yes
Pasini et al. [33]	Yes	Unclear	N/A	Yes	Yes	Yes	Unclear	Yes

Results

Study Selection

**Figure 1:**
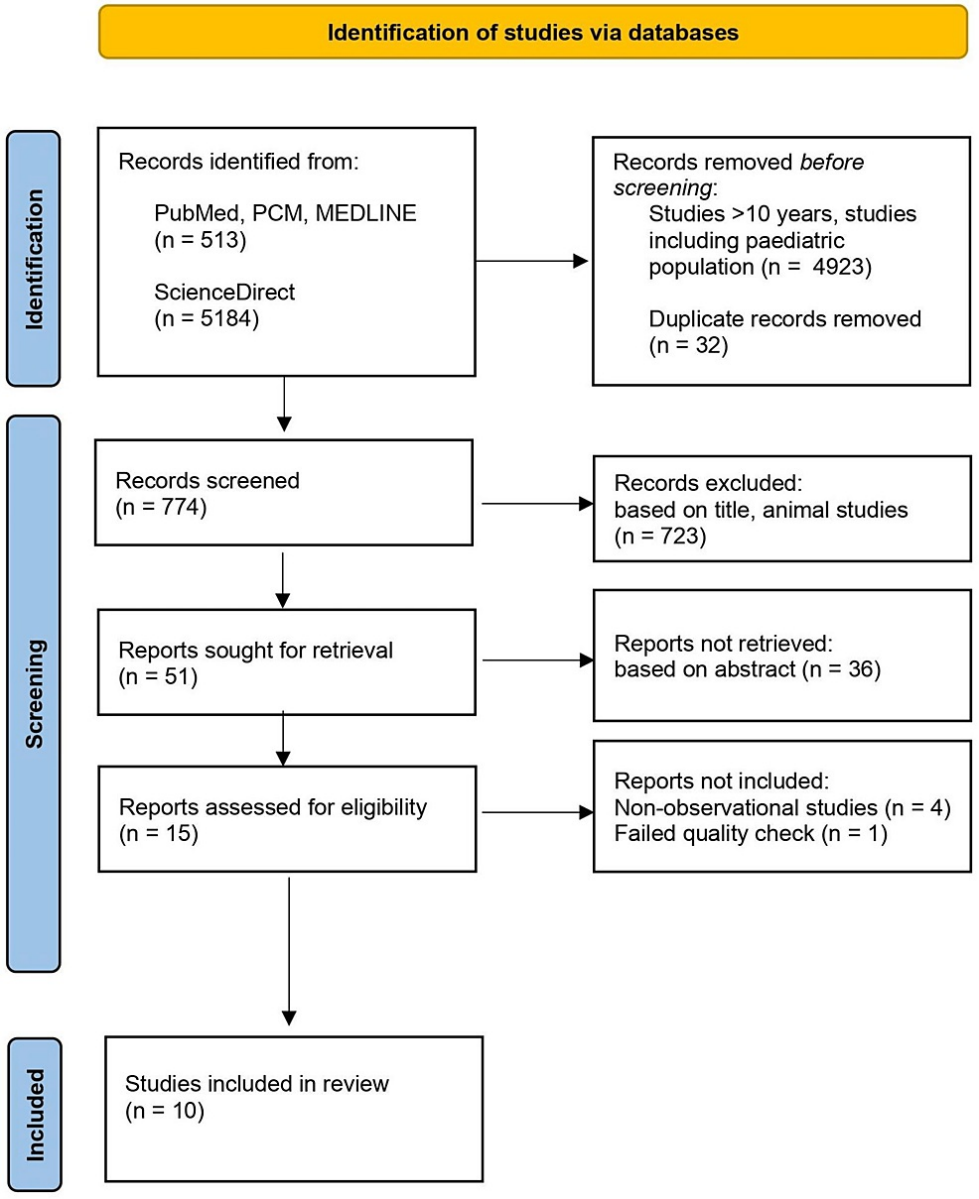
PRISMA flowchart PMC - Pubmed Central, PRISMA - Preferred Reporting Items for Systematic Reviews and Meta-Analyses

Initially, our search included a total of 5697 articles from PubMed, PubMed Central (PMC), Medline, and ScienceDirect databases. After excluding duplicates and articles published more than 10 years ago and focusing on the pediatric population, we included 774 articles for further screening. Screening based on title, abstracts, and inclusion and exclusion criteria excluded 759 articles. We further excluded articles that were non-observational studies and did not meet the criteria during quality appraisal using the JBI tool. Ultimately, 10 articles were selected for review (Figure 1). All the articles were cross-sectional observational studies.

Study Population

The sample size included 705 patients with stable chronic heart failure, acute de novo heart failure, or acute decompensation of chronic heart failure. Patients with both heart failure with reduced ejection fraction and preserved ejection fraction were included to understand the effects of ejection fraction on the gut microbiome. Patients were also included from different stages of heart failure, including New York Heart Association (NYHA) classes I-IV, patients with left ventricular assist device (LVAD), and heart transplant (HT).

Gut Composition

Eight studies used the method of 16S rRNA gene amplification and sequencing to study compositional and functional changes in the microbiome. In contrast, one study used the 16S rDNA gene amplification and sequencing method. The remaining study identified the colonies using colony-forming units (CFU) after culturing on specific agar.

Nine out of 10 articles compared the composition of the gut microbiome in patients with clinically diagnosed heart failure with controls. All these studies concluded a significant difference in both groups' beta diversity (inter-individual diversity). One of 10 studies studied the effects of progressive stages of heart failure (NYHA classes I-IV, LVAD, and HT) on beta diversity.

Functional and Metabolomic Changes

Four of the ten studies analyzed the functional alteration in the gut metagenome, and five of the ten articles analyzed the changes in the fecal and plasma metabolites in patients with heart failure. The pertinent characteristics of the studies have been discussed in Table 3.

**Table 3:** Characteristics of the studies included in the review HFrEF - heart failure with reduced ejection fraction, HFpEF - heart failure with preserved ejection fraction, SCFA - short chain fatty acids, CHF - chronic heart failure, LVEF - left ventricular ejection fraction, LVAD - left ventricular assist device, HT - heart transplant, NYHA - New York Heart Association, ICM - ischemic cardiomyopathy, DCM - dilated cardiomyopathy, TMAO - trimethylamine N-oxide, IS - indoxyl sulfate, HF - heart failure, RAP - right atrial pressure

Authors	Year of publication	Patients (n)	Controls (n)	Inclusion criteria	Results/ conclusion
Beale et al. [24]	2021	26 (HFrEF - 0, HFpEF - 26)	67	HFpEF is confirmed as resting pulmonary capillary wedge pressure >=15 mm Hg or exercise pulmonary capillary wedge pressure >=25 mmHg and an LVEF >50%	Both cohorts significantly differ in alpha-diversity (number of microbes) and Beta-diversity (type and abundance of microbes). Depletion of short-chain fatty acids (SCFAs) producing bacteria in the gut microbiome of patients.
Wang et al. [25]	2021	25	25	Clinically stable patients with CHF	Significant reduction in diversity and complexity of gut microbiota, and serum metabolites in elderly patients with CHF.
Hayashi et al. [26]	2021	22 (HFrEF - 12, HFpEF - 10)	11	Patients admitted for de novo acute decompensated heart failure or acute worsening of chronic heart failure; HFrEF defined as LVEF <=40%, HFpEF defined as LVEF >=50%	Alterations in composition of gut microbial genes involved in the metabolism of essential amino acids in heart failure patients. Reduced plasma levels of total essential amino acids in heart failure patients.
Yuzefpolskaya et al. [27]	2020	452 (HFrEF - 452, HFpEF - 0)	0	Patients >=18 years of age with systolic heart failure	Significant reduction in gut microbial diversity and richness. Elevation of markers of endotoxemia, inflammation, and oxidative stress with the progression of heart failure from NYHA Class I to Class IV. Persistent lower gut diversity and endotoxemia in patients with LVAD and HT, however, they have lower levels of inflammation and oxidative stress.
Cui et al. [28]	2018	53 (HFrEF - 53, HFpEF - 0)	41	Age >18 years, medical history of CHF either from ICM or DCM for more than six months, NYHA classification II to IV, LVEF ≤ 40%	Significant difference in the composition of gut microbiota in CHF patients. Imbalanced gut microbes are involved in metabolizing protective metabolites such as butyrate and harmful metabolites such as trimethylamine N-oxide in CHF patients. Significant change in metabolic features of both fecal and plasma samples from CHF patients.
Katsimichas et al. [29]	2018	28 (HFrEF - 28, HFpEF - 0)	19	Patients with chronic stable heart failure, acute de novo heart failure or acute decompensated chronic heart failure of non-ischemic etiology; HFrEF is defined as LVEF <50%	Significant alterations in gut microbial composition and bacterial gene variations involved in amino acids, carbohydrates, vitamins, and xenobiotic metabolism in patients with heart failure with reduced ejection fraction due to non-ischemic cause.
Hayashi et al. [30]	2018	22 (HFrEF - 12, HFpEF – 10)	11	Age > 18 years, NYHA functional classification II to IV, patients admitted for de novo acute decompensated heart failure or acute worsening of chronic heart failure; HFrEF defined as LVEF <40%, HFpEF defined as LVEF >=50%	Significant alterations in gut microbiome composition and microbiome-related metabolites in patients. Possible correlations between specific bacterial genera and circulating levels of harmful metabolites such as TMAO and IS.
Kamo et al. [31]	2017	22 (HFrEF - 18, HFpEF - 4)	12	Age > 18 years, NYHA functional class II to IV, patients admitted for acute decompensated heart failure or acute exacerbation of chronic heart failure	Significant alterations in gut microbiota in patients with HF. The composition of gut microbiota in heart failure patients varies according to age.
Luedde et al. [32]	2017	20 (HFrEF - 20, HFpEF - 0)	20	Patients with heart failure due to ICM or DCM with LVEF <=35%	Significant reduction in gut microbial diversity in heart failure patients.
Pasini et al. [33]	2015	60	20	Patients with mild CHF (NYHA Class I or II) and moderate to severe CHF (NYHA Class III or IV)	Significant overgrowth of pathogenic bacteria and candida, RAP, and inflammation in patients with CHF; variables more pronounced with moderate to severe CHF patients as compared to mild CHF as determined by the NYHA class. Increased intestinal permeability is associated with clinical disease severity, venous blood congestion, and inflammation.

Discussion

**Figure 2:**
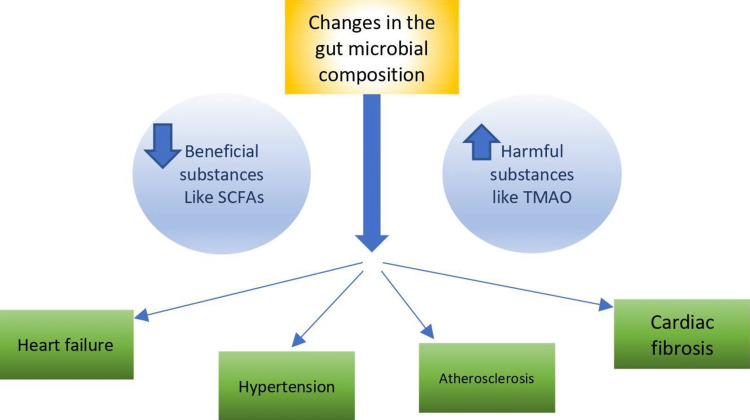
Effects of gut microbiota on the cardiovascular system SCFAs - short-chain fatty acids, TMAO - trimethylamine N-oxide

Interaction between gut microbiota with other organ systems in the body, including the cardiovascular system, has been a recent topic of interest, particularly due to their potential role in the pathogenesis and prognosis of heart failure. The effects of gut microbiota on the cardiovascular system are shown in Figure 2.

Our systematic review aimed to unravel gut microbial diversity, composition alterations, and associated functional and metabolomic changes in patients with chronic heart failure. 

Gut Microbial Richness and Diversity in Heart Failure

A diverse gut microbiome consists of millions of microbial species, each contributing uniquely to maintaining homeostasis and coexisting in a healthy symbiotic relationship with the host gut. Alpha diversity is the total number of species (measure of intra-individual diversity). In contrast, beta diversity is the type and abundance of specific species (measure of inter-individual diversity) present at any time in the gut. In the following few paragraphs, we have described the alterations in gut microbial composition seen in CHF patients and their implications. The characteristics of the sample and the notable changes in the gut microbial composition have been described in Table 4.

**Table 4:** Changes in the gut composition in heart failure patients LVEF - left ventricular ejection fraction, HF - heart failure, rRNA - ribosomal RNA, rDNA - ribosomal DNA, SCFA - short chain fatty acids, LDA - linear discriminant analysis, DCM - dilated cardiomyopathy, ICM - ischemic cardiomyopathy, OTU - operational taxonomic unit

Author	Patients	LVEF %	Age	Method used to evaluate Bacterial DNA from fecal samples	Changes in gut microbial diversity and richness in HF	Elevated relative abundance in HF patients	Reduced relative abundance in HF patients
Beale et al. [24]	Diastolic heart failure	60.5 +/- 5.1	68 +/- 7.5	16S rRNA gene amplification	Significant difference in alpha and beta diversity	-	Ruminococcus, other SCFA-producing bacteria
Wang et al. [25]	Clinically stable patients with chronic heart failure	-	65 +/- 3.17	16s rDNA gene amplification, LEfSe	Significantly reduced fecal microbial diversity and richness in patients with HF. Significant difference in beta diversity.	Ruminococcus gnavus, Escherichia Shigella, Ruminococcaceae UCG 005, Ruminococcaceae UCG 002, Lactobacillus, Atopobium, Romboutsia, Haemophilus, and Klebsiella	-
Hayashi et al. [26]	De novo acute decompensated heart failure or acute exacerbation of chronic heart failure	<= 40 (12), >=50 (10)	72 +/- 18	Whole-genome shot-gun sequencing by Illumina Hiseq 2,500, Linear discriminant analysis (LDA) effect size (LEfSe)	Significant difference in composition of the gut microbiome.	Actinobacteria and Deltaproteobacteria classes; Bifidobacteriales and Desulfovibrionales orders; Bifidobacteriaceae, Porphyromonadaceae and Desulfovibrionaceae families; Bifidobacterium, Gordonibacter, Bilophila and Pseudoflavonifractor genera.	Eubacteriaceae and Succinivibrionaceae families; Eubacterium, Prevotella, Finegoldia and Succinatimonas genera.
Cui et al.[28]	Stable systolic chronic heart failure due to DCM or ICM admitted to hospital	29.79 +/- 6.54	58.08 +/- 13.30	16S rRNA gene amplification	Significant difference in beta diversity	Ruminococcus gnavus, Streptococcus sp, Veillonella sp	Faecalibacterium
Kastimichas et al. [29]	Chronic stable, acute HF/ acute decompensated chronic systolic HF due to non-ischemic causes	25 +/- 9	51 +/- 10	16S rRNA gene amplification	No difference in alpha diversity. Significant difference in beta diversity	Streptococcus sp, Veillonella sp	SMB53
Hayashi et al. [30]	Systolic or diastolic heart failure patients evaluated in decompensated and compensated phases	42 +/- 17	72 +/- 18	16S rRNA gene amplification	No difference in alpha diversity at the genus level. Significant difference in composition of the gut microbiome.	Phylum Actinobacteria, Genus Bifidobacterium	Genus Megamonas
Kamo et al.[31]	Acute decompensated heart failure or acute exacerbation of chronic heart failure	20 +/- 2.2 (12), 43.1 +/- 5.8 (10)	47.4 +/- 2.8 (12), 73.8 +/- 2.8 (10)	16S rRNA gene amplification	No changes in alpha diversity. Greater beta diversity based on weighted and unweighted uniFrac analyses	-	Genus Clostridium and Dorea. Species Eubacterium rectale and Dorea Longicatena.
Luedde et al. [32]	Acute decompensated or stable systolic heart failure due to ICM or DCM	22.3 +/- 2.85	65 +/- 3.17	16S rRNA gene amplification	Significant decrease in alpha (intra-individual) diversity according to Shannon index based on OTU. Significant separation of cases and controls based on Beta diversity (inter-individual) measures.	-	Coriobacteriaceae, Erysipelotrichaceae and Ruminococcaceae families; Blautia, Collinsella, uncl. Erysipelotrichaceae, uncl. Ruminococcaceae genera
Pasini et al. [33]	Mild systolic HF (n=30) and moderate to severe systolic HF (n=30)	39 +/- 1.4 (30), 35 +/- 1.2 (30)	65 +/- 1.3 (30), 63 +/- 1.5 (30)	Stool culture using agar	-	Candida, Campylobacter, Shigella, Salmonella, Yersinia	-

Studies Involving Patients With Systolic and Diastolic Heart Failure

The interplay between the gastrointestinal and cardiovascular systems has been a recent topic of interest for many. Wang et al. found that the overall richness and diversity of bacteria in patients with clinically stable CHF was significantly lower than in controls [25]. They identified the overgrowth of pathogenic bacteria such as *Escherichia Shigella*,* Klebsiella*, and *Haemophilus *as well a significant enrichment of *Ruminococcus gnavus* in CHF patients. *Ruminococcus gnavus* was particularly interesting since it was also elevated in a prior study [28]. It has been shown to possess pro-inflammatory properties in a prior study and, therefore, might contribute to developing a pro-inflammatory state in chronic heart failure and consequently affect its severity and prognosis [34]. While this study further re-enforces the findings of several prior studies, it cannot be ignored that this was also a small-sized study [25]. In addition, the patients were not classified according to the type of heart failure, i.e., systolic or diastolic. Although a prior study conducted by Hayashi et al. has refuted the role of ejection fraction in dysbiosis, further large-scale longitudinal studies are needed to exclude the effect of LVEF% on gut dysbiosis [26].

A recent study has described the most notable change in the gut microbiome in HF patients as the depletion of *Eubacterium *and *Prevotella *on network analysis [26]. These genera play a significant role in the biosynthesis of essential amino acids in the gut. The most important merit of the study, apart from it being recent, was that it included patients with heart failure with reduced ejection fraction (HFrEF) as well as heart failure with preserved ejection fraction (HFpEF) and identified that the alterations in the composition of gut microbiome did not vary significantly in those groups as compared to the controls. It could be understood from this that ejection fraction does not play a significant role in the gut dysbiosis seen in patients with CHF.

A small-sized study identified that patients with HF had no differences in alpha diversity; however, they had a decreased representation of *Eubacterium rectale* and *Dorea longicatena*. They further studied that the older patients had a lower proportion of *Bacteroidetes *and a larger proportion of *Proteobacter *than younger patients with heart failure. In addition, older patients also had elevated *Lactobacillus *and reduced *Faecalibacterium prausnitzii* and *Clostridium clostridioforme* in the feces. Depleting *Eubacterium rectale* and *Faecalibacterium prausnitzii* leads to depletion of butyrate production in the gut affecting the gut integrity and creating a pro-inflammatory state in older patients with HF. While they have elucidated the relationship of dysbiosis with age, the major limitation of their study remains the potential confounding effect of aging as a natural process, medications, diet, and comorbidities on the gut microbiome. The study could not establish a temporal relationship between aging in HF patients and gut dysbiosis. Therefore, long-term longitudinal studies are recommended to understand specific aging alterations to create unique treatment plans based on the patient's age [31].

A study also found a significant reduction in the total number of microbes in HF patients, apart from identifying a non-significant overgrowth of pathogenic bacteria in the gut. There was a significant reduction of *Blautia*, *Collinsella*, unclassified *Erysipelotrichaceae*, and unclassified *Ruminococcaceae genera*. Furthermore, they proposed that the depletion of *Collinsella* was specific to HF due to its depletion even in patients with HF and comorbid diabetes mellitus or ischemic heart disease [32]. Blautia was also recently studied to possess anti-inflammatory properties. Hence, its demonstrated depletion might contribute to a pro-inflammatory state that further worsens the severity and progression of HF. A constellation of these changes could be a specific finding of HF and must further be explored in large-scale, multi-centric longitudinal studies.

Studies Involving Patients With Only Systolic Heart Failure

Cui et al. studied the samples' beta diversity (based on the Bray Curtis distances). They found that the microbiome composition was significantly different among HFrEF patients and independent of the usage of PPI or statins. However, there was no difference in the beta diversity between the ischemic cardiomyopathy (ICM) and dilated cardiomyopathy (DCM) patients, concluding that the changes in the beta diversity are independent of the etiology of HFrEF. The essential characteristics were increased representation of the *Ruminococcus gnavus* and decreased representation of Faecalibacterium prausnitzii, as identified in the feces [28]. *Faecalibacterium prausnitzii *has been studied to possess an anti-inflammatory property due to its role in butyrate production [35]. Since butyrate plays an essential role in preventing inflammation in the body and damage to the gut, reducing butyrate-producing bacteria contributes to the pro-inflammatory state found in CHF. This interplay between bacteria affecting the butyrate homeostasis in the body has been hypothesized to contribute to the functional and metabolic changes in CHF. Hence, we propose further studies to identify these derangements as therapeutic targets to prevent inflammation and progression seen in CHF. While this was a multi-center study focusing on patients with systolic dysfunction, it primarily involved hospitalized patients with poor cardiac function (NYHA classes III and IV) and hence, might have missed the representation of stable patients with systolic HF. Moreover, the patients' diet and exercise regimens were not considered.

Another study that evaluated the diversity in HFrEF patients identified that patients with systolic HF did not have a significant drop in the total number of species (alpha diversity) comparable to the controls; however, they have notable alterations in the composition of the microbiome. They observed an increased relative abundance of phylum *Actinobacteria *and genus *Bifidobacterium *and a reduced abundance of genus *Megamonas *[30]. They proposed that the interplay between these bacteria contributed to developing a pro-inflammatory state in systolic HF; similar changes had been previously observed in patients with Behcet's disease [36]. However, this study had several limitations, such as a smaller sample size, single-center study and failure to exclude confounding effects of diet and exercise on the gut. Another study that evaluated the alterations in gut microbial composition in HFrEF patients inferred that although the alpha diversity did not vary significantly among groups, the beta diversity varied even after adjusting for age, renal function and medication consumption [29]. There was an enrichment of Streptococcus and Veillonella sp. and a reduction of SMB53 in HFrEF patients compared to the control group.

A study conducted in patients with systolic heart failure identified that the patients with HFrEF have a significant overgrowth of pathogenic organisms such as Campylobacter, Shigella, Salmonella, Yersinia, and candida [33]. Similarly, a study identified overgrowth of pathogenic Enterobacteriaceae, especially Escherichia/ Shigella clusters; however, the levels did not reach significance [32]. HF patients also had an increased intestinal permeability (IP), right atrial pressure (RAP), and C-Reactive protein (CRP). Pasini et al. were the first to conclude that the pathogenic gut flora overgrowth, IP, RAP, and CRP were interrelated. They further mentioned that these measurements increased with the severity of HF, as per the NYHA classes [33]. High RAP leads to increased IP through decreased intestinal blood circulation and inflammation, causing an overgrowth of pathogenic organisms [37] . This pathogenic overgrowth ultimately impairs the production of essential intestinal metabolites such as short chain fatty acids (SCFA), thus creating a pro-inflammatory state and affecting the cardiovascular function and CHF progression [38]. Whether or not the re-establishment of gut microbiota prevents the progression of CHF is a matter of further studies.

Yuzefpolskaya et al. conducted a large-scale study to identify the gut composition variation across varying stages of heart failure - NYHA Class I-IV, LVAD, and HT. They observed a decrease in the gut diversity across stages from NYHA class I to class IV. Moreover, the diversity remained low among patients with LVAD and HT. This diversity depletion was primarily due to a subset of taxa that possess anti-inflammatory features [27].

Studies Involving Patients With Only Diastolic Heart Failure

While many studies had been conducted on HFrEF patients to evaluate gut dysbiosis, the study conducted by Beale et al. was unique since it included only patients with diastolic heart failure, aiming at identifying gut microbial dysbiosis in patients with HFpEF. They concluded that the patients with HFpEF had varying alpha as well as beta diversity compared to the controls. They observed that the major contributor to the variation in beta diversity was the significant depletion of SCFA-producing microbes, particularly *Ruminococcus *[24]. The interplay between SCFAs, the cardiovascular system, and the body's immune system has been studied recently. Depletion of SCFAs contributes to the pathogenesis and prognosis of HFpEF by affecting numerous factors in the body, such as insulin resistance, diabetes control, obesity, hypertension, and myocardial hypertrophy and fibrosis. Beale et al., therefore, proposed that the SCFA-producing bacteria in the gut could be potential targets for the prevention of the progression of HFpEF.

Furthermore, this study also observed that the changes in beta diversity in patients with HFpEF were independent of dietary factors [24]. However, this was another small-sized study that only included patients with HFpEF. Therefore, understanding the effect of dietary factors on the gut cannot be applied to all patients with HF.

Functional and Metabolomic Alterations in Heart Failure

Along with studying the gut microbiome composition in CHF patients, it is also pertinent to identify the implications of those changes on the functional expression of genes and metabolic pathways to understand the effect of dysbiosis in greater detail. Several studies have extrapolated their compositional findings to identify gene and metabolic expression changes, as summarized in Table 5.

**Table 5:** Functional and metabolomic changes in heart failure patients HF - heart failure, ELISA - enzyme-linked immunosorbent assay, LC-MS/MS - liquid chromatography with tandem mass spectrometry, IL - interleukin, TNF - tumor necrosis factor, CRP - C-reactive protein, CE-TOF MS - capillary electrophoresis time of flight mass spectrometer, LAL - limulus amebocyte lysate, ET - endothelin, LVAD - left ventricular assist device, LPS - lipopolysaccharides, HT - heart transplant, TMA-lyase - trimethylamine-lyase, PICRUSt - phylogenetic investigation of communities by reconstruction of unobserved states, HFpEF - heart failure with preserved ejection fraction

Author	Methods used to identify alterations in the metagenome	Functional alterations in microbiota metagenome	The method used to identify metabolic alteration in plasma/ serum/ fecal sample	Plasma/ serum metabolic changes in HF patients	Fecal metabolic changes in HF patients
Wang et al. [25]	-	-	ELISA, LC-MS/MS	Significant elevation of IL-6, IL-8, TNF-a and CRP levels in serum. Significant reduction of IL-10 levels in serum.	-
Hayashi et al. [26]	HMP Unified Metabolic Analysis Network	An increased abundance of gut microbial genes responsible for degrading essential amino acids, including branch-chain amino acids and histidine. A decreased abundance of gut microbial genes responsible for the biosynthesis of essential amino acids, including branch-chain amino acids and histidine.	CE-TOF MS, LC-MS/MS	Reduced plasma levels of total essential amino acids, histidine, and alanine.	-
Yuzefpolskaya et al. [27]	-	-	High sensitivity (0.3mg/L) particle enhanced turbidimetric assay, high sensitivity Enzyme-Linked Immunoassay (ELISA), LAL chromogenic endotoxin quantitation kit, LC-MS/MS	Significant elevation of markers of inflammation (CRP, IL-6, TNF-a, ET-1 and adiponectin) and oxidative stress (isoprostane) in patients with Class IV HF as compared to Classes I-III. They were reduced in patients with LVAD and post-HT compared to Class IV HF. Among markers of endotoxemia, LPS was elevated in patients with Class IV HF, LVAD and HT. sCD14 was elevated in patients with Class IV HF and LVAD but not in patients with HT.	-
Cui et al. [28]	Functional annotations of the metagenome to the KEGG modules	Upregulation of genes for choline TMA-lyase. Upregulation of genes for lipopolysaccharide biosynthesis, tryptophan and lipid metabolism. Downregulation of genes for synthesis and transport of amino acids, nucleotide sugar biosynthesis, iron transport system and short-chain fatty acid metabolism. Downregulation of genes for butyrate acetoacetate CoA transferase.	LC/MS	Increased 49 metabolites such as sphingosine 1-phosphate. Decreased 59 metabolites such as ricinoleic acid	Increased two metabolites such as para-Tolyl octanoate. Decreased 206 metabolites such as niacin, cinnamic acid and orotic acid.
Katsimichas et al. [29]	PICRUSt.	Significant differences in gene families involved in the metabolism of amino acids, carbohydrates, vitamins, and xenobiotics.	-	-	-
Hayashi et al.[30]	PICRUSt.	An increased abundance of microbial TMA-lyase and tryptophanase genes in decompensated heart failure patients	CE-TOF MS	Reduced concentration of indoxyl sulfate and indoxyl sulfate to tryptophan ratio in decompensated heart failure patients. Reduced concentration of tryptophan and higher indoxyl sulfate to tryptophan ratio in patients with compensated HFpEF.	-

Most recently, two studies have focused on the topic of interest. Wang et al. studied the serum concentrations of the markers of inflammation (IL-6, IL-8, TNF-a, and IL-10) in patients with stable chronic heart failure [25]. Earlier, it has been studied that an overgrowth of pathogenic gut flora and increased intestinal permeability are interrelated [33]. Inflammation causes leakage of products from the gut microbiota to seep into the circulation and enters the bloodstream. Based on this understanding, Wang et al. observed an increased concentration of pro-inflammatory markers such as IL-6, IL-8, and TNF-a in patients compared to controls. They also observed a decreased level of IL-10 in their serum. CRP levels were also significantly elevated in the patients [25]. This finding further consolidates the notion of increased intestinal permeability in CHF due to gut dysbiosis and the role of inflammation in the pathogenesis of heart failure. All the patients included in this study were elderly and clinically stable. Therefore, the findings of this study cannot be applied to young patients or clinically unstable patients with an exacerbation of heart failure. Moreover, the LVEF% of the patients has not been clearly defined.

A similar study conducted primarily in patients with systolic HF across varying stages concluded that the levels of inflammation such as IL-6, CRP, adiponectin, endothelin, and TNF-a, and oxidative stress, such as isoprostane increased along with the severity of heart failure. Eventually, they decreased in LVAD and HT patients [27]. Therefore, it could be understood that inflammation plays a significant role in the progression of systolic heart failure. After insertion of the assist device and heart transplantation, as the ejection fraction improved, the increased circulation in the gut improves dysbiosis, and consequently, the inflammation reduces. On the other hand, they also observed that the biomarker of endotoxemia (LPS) remained elevated after LVAD and HT, whereas sCD14 remained elevated only after LVAD and decreased in HT [27]. These findings elucidated the interplay between the reduced ejection fraction, increased gut permeability, gut dysbiosis, and inflammation.

In 2021, Hayashi et al. investigated the relationship between gut dysbiosis and changes in the metabolism of amino acids. At the metagenome level, they observed an increased abundance of genes responsible for degradation and decreased abundance of genes responsible for the biosynthesis of essential amino acids. As a result, the levels of essential amino acids, including branch-chain amino acids, alanine, and histidine, are in the blood of patients with heart failure. They also established a positive correlation between microbial essential amino acid biosynthesis genes and plasma branch-chain amino acid levels [26]. Although the study revealed an association between dysbiosis and metabolic alterations, it failed to establish a temporal causal relationship between dysbiosis-driven metabolic alterations and the development of heart failure. Earlier, a similar study also reported a significant difference in the abundance of genes responsible for the metabolism of amino acids, xenobiotics, carbohydrates, and vitamins [29].

The role of TMAO on cardiovascular health has been widely studied recently. Upregulation of genes for choline trimethylamine-lyase (TMA-lyase) and Llpopolysaccheride (LPS) biosynthesis was observed in a cohort of patients with HFrEF [28]. TMA-lyase is an enzyme responsible for the generation of TMAO. TMAO has directly been linked with the progression of CHF through its effects on the kidney, causing renal tubulointerstitial fibrosis and dysfunction [39]. In addition, the downregulation of genes for butyrate acetoacetate CoA transferase, synthesis and transport of amino acids, nucleotide sugar biosynthesis, iron transport system, and short-chain fatty acid metabolism. Butyrate is anti-inflammatory and has protective effects on the intestines. Downregulation of genes responsible for butyrate synthesis decreases the butyrate concentration in the host, promoting a pro-inflammatory state that could further worsen the severity of heart failure. The metabolomic analysis also revealed an imbalance of several metabolites, the most notable being the increase of sphingosine 1-phosphate [28]. Sphingosine 1-phosphate has been shown to cause cardiac dysfunction and remodeling along with an active role in several other pathological processes affecting the cardiovascular system [40].

In patients with decompensated heart failure, an increased expression of TMA-lyase and tryptophanase genes was observed [30]. TMAO would be elevated in these patients and might mediate the progression of heart failure. Moreover, they observed a positive correlation between the abundance of *Escherichia/ Shigella* and levels of indoxyl sulfate and TMAO. Indoxyl sulfate is a microbial-dependent uremic toxin that is known to harm the cardiovascular system. Upregulation of the tryptophanase genes in the microbiota in the decompensated phase causes indoxyl sulfate production in the host. They also proposed an inverse correlation between the abundance of genus Bifidobacterium and the plasma indoxyl sulfate levels and that it has a cardioprotective effect in patients with HF [30]. 

Limitations

Firstly, most of the studies included were single-centered small-sized studies involving a small patient population. The gut microbiota is affected by many factors, including the patient's age, comorbid conditions, diet, medications, and exercise. Dietary and exercise history was not considered in most of the included studies. Moreover, there was a significant difference in the medication usage of the patients and the control subjects. None of these studies identified a temporal relationship between gut dysbiosis and heart failure. Therefore, large-scale, multi-center longitudinal studies must understand whether they have a causal relationship. 

## Conclusions

There is a significant alteration in the richness and composition of the gut microbiome in patients with heart failure. The composition further varies according to the age, severity, and stage of heart failure. Gut dysbiosis further influences the metabolic homeostasis of the host. It creates an imbalance of important metabolites in plasma and feces, such as TMAO, indoxyl sulfate, amino acids, and inflammatory markers. These metabolic disturbances promote a pro-inflammatory state that further affects the severity and progression of heart failure. These are the fecal and plasma biomarkers of heart failure, and they could prove to be crucial targets for preventing the development and prognosis of heart failure. We propose further large-scale longitudinal studies to identify a temporal relationship between gut dysbiosis and heart failure, if any exists.

## References

[1] Heidenreich PA, Albert NM, Allen LA (2013). Forecasting the impact of heart failure in the United States: a policy statement from the American Heart Association. Circ Heart Fail.

[2] Shreiner AB, Kao JY, Young VB (2015). The gut microbiome in health and in disease. Curr Opin Gastroenterol.

[3] Kloos WE, Musselwhite MS (1975). Distribution and persistence of Staphylococcus and Micrococcus species and other aerobic bacteria on human skin. Appl Microbiol.

[4] Luckey TD (1972). Introduction to intestinal microecology. Am J Clin Nutr.

[5] Paster BJ, Boches SK, Galvin JL (2001). Bacterial diversity in human subgingival plaque. J Bacteriol.

[6] Robinson CJ, Bohannan BJ, Young VB (2010). From structure to function: the ecology of host-associated microbial communities. Microbiol Mol Biol Rev.

[7] Frank DN, St Amand AL, Feldman RA, Boedeker EC, Harpaz N, Pace NR (2007). Molecular-phylogenetic characterization of microbial community imbalances in human inflammatory bowel diseases. Proc Natl Acad Sci USA.

[8] Wen L, Ley RE, Volchkov PY (2008). Innate immunity and intestinal microbiota in the development of type 1 diabetes. Nature.

[9] Shreiner A, Huffnagle GB, Noverr MC (2008). The "Microflora Hypothesis" of allergic disease. Adv Exp Med Biol.

[10] Wu S, Rhee KJ, Albesiano E (2009). A human colonic commensal promotes colon tumorigenesis via activation of T helper type 17 T cell responses. Nat Med.

[11] Arpaia N, Campbell C, Fan X (2013). Metabolites produced by commensal bacteria promote peripheral regulatory T-cell generation. Nature.

[12] Atarashi K, Tanoue T, Oshima K (2013). Treg induction by a rationally selected mixture of Clostridia strains from the human microbiota. Nature.

[13] Atarashi K, Tanoue T, Shima T (2011). Induction of colonic regulatory T cells by indigenous Clostridium species. Science.

[14] Furusawa Y, Obata Y, Fukuda S (2013). Commensal microbe-derived butyrate induces the differentiation of colonic regulatory T cells. Nature.

[15] Narushima S, Sugiura Y, Oshima K, Atarashi K, Hattori M, Suematsu M, Honda K (2014). Characterization of the 17 strains of regulatory T cell-inducing human-derived Clostridia. Gut Microbes.

[16] Smith PM, Howitt MR, Panikov N (2013). The microbial metabolites, short-chain fatty acids, regulate colonic Treg cell homeostasis. Science.

[17] Enaud R, Prevel R, Ciarlo E, Beaufils F, Wieërs G, Guery B, Delhaes L (2020). The gut-lung axis in health and respiratory diseases: a place for inter-organ and inter-kingdom crosstalks. Front Cell Infect Microbiol.

[18] Carabotti M, Scirocco A, Maselli MA, Severi C (2015). The gut-brain axis: interactions between enteric microbiota, central and enteric nervous systems. Ann Gastroenterol.

[19] Scher JU, Sczesnak A, Longman RS (2013). Expansion of intestinal Prevotella copri correlates with enhanced susceptibility to arthritis. Elife.

[20] Le Chatelier E, Nielsen T, Qin J (2013). Richness of human gut microbiome correlates with metabolic markers. Nature.

[21] Karlsson FH, Tremaroli V, Nookaew I (2013). Gut metagenome in European women with normal, impaired and diabetic glucose control. Nature.

[22] Wang Z, Klipfell E, Bennett BJ (2011). Gut flora metabolism of phosphatidylcholine promotes cardiovascular disease. Nature.

[23] Page MJ, McKenzie JE, Bossuyt PM (2021). The PRISMA 2020 statement: an updated guideline for reporting systematic reviews. BMJ.

[24] Beale AL, O'Donnell JA, Nakai ME (2021). The gut microbiome of heart failure with preserved ejection fraction. J Am Heart Assoc.

[25] Wang Z, Cai Z, Ferrari MW, Liu Y, Li C, Zhang T, Lyu G (2021). The correlation between gut microbiota and serum metabolomic in elderly patients with chronic heart failure. Mediators Inflamm.

[26] Hayashi T, Yamashita T, Takahashi T (2021). Uncovering the role of gut microbiota in amino acid metabolic disturbances in heart failure through metagenomic analysis. Front Cardiovasc Med.

[27] Yuzefpolskaya M, Bohn B, Nasiri M (2020). Gut microbiota, endotoxemia, inflammation, and oxidative stress in patients with heart failure, left ventricular assist device, and transplant. J Heart Lung Transplant.

[28] Cui X, Ye L, Li J (2018). Metagenomic and metabolomic analyses unveil dysbiosis of gut microbiota in chronic heart failure patients. Sci Rep.

[29] Katsimichas T, Ohtani T, Motooka D (2018). Non-ischemic heart failure with reduced ejection fraction is associated with altered intestinal microbiota. Circ J.

[30] Hayashi T, Yamashita T, Watanabe H (2018). Gut microbiome and plasma microbiome-related metabolites in patients with decompensated and compensated heart failure. Circ J.

[31] Kamo T, Akazawa H, Suda W (2017). Dysbiosis and compositional alterations with aging in the gut microbiota of patients with heart failure. PLoS One.

[32] Luedde M, Winkler T, Heinsen FA (2017). Heart failure is associated with depletion of core intestinal microbiota. ESC Heart Fail.

[33] Pasini E, Aquilani R, Testa C (2016). Pathogenic gut flora in patients with chronic heart failure. JACC Heart Fail.

[34] Joossens M, Huys G, Cnockaert M (2011). Dysbiosis of the faecal microbiota in patients with Crohn's disease and their unaffected relatives. Gut.

[35] Sokol H, Pigneur B, Watterlot L (2008). Faecalibacterium prausnitzii is an anti-inflammatory commensal bacterium identified by gut microbiota analysis of Crohn disease patients. Proc Natl Acad Sci USA.

[36] Shimizu J, Kubota T, Takada E (2016). Bifidobacteria abundance-featured gut microbiota compositional change in patients with Behcet's disease. PLoS One.

[37] Arutyunov GP, Kostyukevich OI, Serov RA, Rylova NV, Bylova NA (2008). Collagen accumulation and dysfunctional mucosal barrier of the small intestine in patients with chronic heart failure. Int J Cardiol.

[38] Gibson GR, Macfarlane GT, Cummings JH (1993). Sulphate reducing bacteria and hydrogen metabolism in the human large intestine. Gut.

[39] Tang WH, Wang Z, Kennedy DJ (2015). Gut microbiota-dependent trimethylamine N-oxide (TMAO) pathway contributes to both development of renal insufficiency and mortality risk in chronic kidney disease. Circ Res.

[40] Zhang F, Xia Y, Yan W (2016). Sphingosine 1-phosphate signaling contributes to cardiac inflammation, dysfunction, and remodeling following myocardial infarction. Am J Physiol Heart Circ Physiol.

